# Struggles and Joys: A Mixed Methods Study of the Artefacts and Reflections in Medical Student Portfolios

**DOI:** 10.5334/pme.1029

**Published:** 2024-01-05

**Authors:** Jenny McDonald, Wendy Hu, Sylvia Heeneman

**Affiliations:** 1 Translational Health Research Institute, School of Medicine, Western Sydney University, South Penrith, Australia; 2 School of Health Profession Education, Maastricht University, the Netherlands

## Abstract

**Introduction:**

Portfolios scaffold reflection on experience so students can plan their learning. To elicit reflection, the learning experiences documented in portfolios must be meaningful. To understand what experiences first- and second-year medical students find meaningful, we studied the patterns in the artefacts chosen for portfolios and their associated written reflections.

**Methods:**

This explanatory mixed methods study of a longitudinal dataset of 835 artefacts from 37 medical student’ portfolios, identified patterns in artefact types over time. Mixed model logistic regression analysis identified time, student and curriculum factors associated with inclusion of the most common types of artefacts. Thematic analysis of participants’ reflections about their artefacts provided insight into their choices. Interpretation of the integrated findings was informed by Transformative Learning (TL) theory.

**Results:**

Artefact choices changed over time, influenced by curriculum changes and personal factors. In first year, the most common types of artefacts were Problem Based Learning mechanism diagrams and group photos representing classwork; in second year written assignments and ‘selfies’ representing social and clinical activities. Themes in the written reflections were Landmarks and Progress, Struggles and Strategies, Connection and Collaboration, and Joyful Memories for Balance. Coursework artefacts and photographic self-portraits represented all levels of transformative learning from across the curriculum.

**Conclusions:**

Medical students chose artefacts to represent challenging and/or landmark experiences, balanced by experiences that were joyful or fostered peer connection. Novelty influenced choice. To maximise learning students should draw from all experiences, to promote supported reflection with an advisor. Tasks should be timed to coincide with the introduction of new challenges.

## Introduction

Portfolios scaffold reflection on experience so students can recognise achievements and address gaps in knowledge and skills [1,2,3,4,5]. This supports competency-based and student-centred learning, two priorities of many modern medical programs [6,7]. To achieve this aim, portfolios should contain evidence of experiences relevant to students’ learning [3,4,8]. However, students report that portfolio compilation and reflection tasks are time-consuming and feel contrived and that portfolios are unhelpful for their learning [2,9,10]. Despite the recommendation that students be given autonomy in selecting evidence [9], little attention has been given to the what types of evidence students find meaningful and how the evidence chosen impacts portfolio-based learning.

Portfolio design and intended purpose determines the evidence collected in portfolios [3,11,12]. When portfolios are designed in part, or wholly for assessment, the student’s attention is shifted towards fulfilling the portfolio assessment requirements, and away from their own perception of progress. This drives the inclusion of assessments of learning such as formal assessment tasks [12] or achievements in clinical settings [3,11,13]. Successes are more likely to be included than mistakes or negative feedback [3], meaning students miss the opportunity to learn from failures. When portfolios are designed for promoting reflection, students respond with the requirements of ‘reflecting’ [14]. The quality of their reflections improves when students reflect on meaningful experiences [8]. Regardless of the purpose and context of a portfolio, it seems likely inclusion of meaningful experiences will increase the relevance of the portfolio to the student’s learning, to support learning.

The link between meaningful experiences and learning is critical reflection. According to Transformative Learning (TL) theory [15,16,17], experience is central to learning. An experience that creates disequilibrium triggers critical reflection on previously held assumptions, knowledge, and values enabling changes in perspective, values, and behaviour [6,18]. For students entering medicine, academic workload and a new culture, peer group and identity may create disequilibrium. However, the perception of disequilibrium is shaped by prior experiences, social context and culture, emotions and relationships [16] and critical reflection may be supported or challenged through dialogue and interaction with peers [19] and mentors [20]. The confluence of personal and social factors means responses to experience are idiosyncratic. For one student an experience is transformative; for another the experience is unremarkable.

When students reflect on experience, Sterling describes three possible levels of learning based on TL theory: confirmative where new knowledge or experience fits existing assumptions; reformative where critical reflection challenges and changes perspective, and transformative learning where critical reflection in the context of a disorientating experience shifts perspective, understanding and interaction with the world. When there is no reflection, there is ‘zero learning’ [21]. Portfolios support learning because the collected artefacts act as souvenirs of experience to prompt reflection on learning.

To explore what experiences that students find meaningful for their learning during the first and second years of a medical program, we studied the types of artefacts included in portfolios, and the experiences they represented. We explored the reflections associated with the artefacts to understand the meanings attributed to the experiences.

Our research questions for our mixed methods study were:

1. What artefacts do medical students include in their portfolios?
2. What curriculum and participant factors predict artefact choices?
3. How do student’ reflections about their portfolio artefacts explain the significance of learning experiences represented by the artefacts?

## Methods and materials

This longitudinal mixed methods study used an explanatory sequential design [22]. We followed JARS-Mixed Methods Article Reporting Standards [23]. Mixed methods research is useful to explore and explain complex phenomena through the synthesis of complementary analyses of data [24]. The initial quantitative phase identified patterns in artefact choices over time and associations between these patterns, and participant and curriculum factors to answer our first two research questions.

The qualitative phase aimed to explain the quantitative findings through a constructionist approach, in that we drew understanding from the students’ written descriptions of their experiences, and the ascribed relevance of the experiences for their learning. Our interpretation of the participants’ reflections was influenced by our experience, values and perspectives [25]. The qualitative phase addressed our third research question. (See Appendix 1 for a flowchart of our study design.)

### Context

The setting for this research is Western Sydney University (WSU), a 5-year undergraduate integrated and competency-based medical program located in an outer metropolitan district of Sydney, Australia. Students are drawn from diverse backgrounds, including direct entry and graduate entry students, migrant, Indigenous, rural, and international. The first two years includes teaching in biomedical sciences and research skills, professionalism, and introductory clinical skills.

At WSU the portfolio is designed to support reflection on learning experiences, and study planning. As an assessment task, first and second-year students prepare presentations about their progress with a learning plan as the basis for the interview with an academic advisor – twice in first year and once in second year. The presentations are created using MyKnowledgeMap (MKM) e-portfolio software and must include two artefacts supported by a descriptive reflection, to represent each of the four curriculum domains (patient care, community health, personal and professional development, and the scientific basis of medicine). Students are instructed in workshops and a handbook to include artefacts of experiences which helped them improve or master some knowledge or a skill or enabled them to solve a problem, to demonstrate what they ‘have learned or still needed to learn. For the accompanying written reflection students were asked to describe how the experience was relevant to the learning outcome for that curriculum domain. Advisors coach students to reflect on their experiences and refine their learning plan. This assessment task must be completed to a satisfactory standard.

### Quantitative data analysis

#### Sample and Procedures

A stratified sample of 37 participants was drawn from the 2020 Year 1 cohort of 137 students by an assistant who was not a member of the teaching staff or research team. The sample size was determined to ensure combinations of participant factors were available, anticipating the probability of least likely units was 1 in 10 [26]. Our choice of independent variables was based on factors likely to influence learning [27], and perceptions of experience according to TL theory [17]. The factors were: gender, prior study experience, academic achievement at the end of first year and domestic or international status. Participant demographic data were collected from university administrative records. To ensure the sample was representative of the cohort for the purpose of the quantitative analysis, the proportions of these factors were compared with those of the cohort. (See Table 1).

**Table 1:** Sample & Cohort Characteristics.


	SAMPLE n = 37	COHORT n = 137	DIFFERENCE

	n (%)	n (%)	

Gender			

Male	20 (54)	84 (61)	NS*

Female	17 (46)	53 (39)	

Prior study			

School leavers	23 (62)	63 (46)	NS*

Prior university study	18 (38)	46 (34)	

Unknown		28 (20)	

Residency			

Domestic	29 (78)	110 (80)	

International	8 (22)	27 (20)	NS*

Mean age at enrolment (range)	21.4 (18–49)	20.5 (17–49)	NS**

Academic performance end of first year	n = 37	n = 137	NS*

Fail	3 (8.1)	14 (10.2)	

Pass	16 (43.2)	73 (53.3)	

High performance (Credit, Distinction or High Distinction)	18 (48.6)	50 (36.5)	

Academic performance 2nd year	n = 34	n = 118	NS*

Fail	2 (5.9)	7 (5.9)	

Pass	17 (50)	57 (48.3)	

High performance (Credit, Distinction or High Distinction)	15 (44)	54 (45.8)	


*Pearson Chi- Square **Independent-Samples Mann-Whitney U Test.

Content analysis was conducted following Krippendorf’s guidelines [26] of all portfolio artefacts from the participants’ interview presentations at three time points in first and second year. The presentation artefacts were de-identified prior to coding. Artefacts were coded for format (text, image, and video), curriculum subject and the curricular or extra-curricular activity they represented. The participant’s description of each artefact was used when necessary to inform coding.

Coding categories and definitions were developed iteratively through research team review to create mutually exclusive and well-defined codes. A sample of artefacts was double-coded by JM and WH to ensure reliability and validity [26], to establish the final codebook. Once a final codebook was developed, JM coded the data independently.

The full dataset of codes was uploaded into the Statistical Package for Social Sciences (SPSS) version 27 (SPSS Inc., Chicago, Illinois). Descriptive analysis determined the frequency and proportions of artefact types and learning activities at different time points. Mixed model binary logistic regression models were built, using the participant as a random factor. The four most frequently chosen types of artefacts were chosen as the outcome. The independent variables: participant gender, prior study experience, academic performance, and international or domestic status were sequentially excluded from the models for each outcome variable until a significant model of best fit was created. Statistical significance was defined as P < 0.05.

### Qualitative data analysis

#### Sample and Procedures

The sample used for the quantitative analysis provided the diversity of perspectives suitable for the qualitative stage of our study.

The de-identified interview presentations were uploaded into NVivo Version 12 (QSR International, Location). The ‘meaning and meaningfulness’ of the experiences represented by the artefacts was explored using reflexive thematic analysis of the reflections associated with each artefact [28,29].

All reflections were read by JM to become familiarised with the data. Initial deductive coding of the participants’ reflections was undertaken, framed by transformative influences. That is, the triggering experience, personal response (assumptions, emotions) context (social relationships, dialogue) and outcomes (changes in perspective, self-awareness, or behaviour). These codes were confirmed with re-reading, note-taking, refinement of the coding and comparison of coding between cases and time periods. Through researcher team discussion and consensus, codes were combined into categories with shared meaning and outcomes. Review of the coded data and relationships between the codes, allowed generation of themes that best encapsulated the patterns in artefact choices as described in the participants’ reflections.

### Integration of the findings

Integration of the findings was achieved through research team discussion, and the development of a joint display demonstrating how patterns in our qualitative findings, related to and explained the patterns in our quantitative findings [30]. TL theory [17,21] informed interpretation of our integrated findings.

#### Reflexivity

Two of the authors (JM, WH) are medically trained academics within WSU medical program and SH is a medical educator with a background in biomedical science from an overseas medical school, providing different perspectives and interpretations on participant context, evidence choices and reflections. All authors are experienced medical educators and qualitative researchers. Our different perspectives, shaped through personal experience, knowledge of the literature and our roles and status as educators in different contexts, were reconciled through regular discussion [31].

#### Ethics

Ethics approval was granted by Western Sydney University Human Research Ethics Committee ID No H9989, amendment 8385.

## Results

### Quantitative analysis

#### Sample

The 37 participants prepared a total of 109 presentations for three interviews at Time 1 (May 2020, early in first year), Time 2 (September 2020, towards the end of first year) and Time 3 (July 2021, mid-second year). The presentations included 767 artefacts with a reflection and, and 68 reflections without an artefact.

There were no significant differences between the participants and students from the same cohort not included in the study. This confirmed our sample was representative of the cohort for the characteristics used in the analysis (See Table 1).

#### Descriptive analysis of artefacts

The artefacts were mostly drawn from curriculum-based activities. The presentations were all unique with diverse choices in artefacts in terms of format, context, and activity.

Textual artefacts accounted for 48.1% of presentation entries. The most common ***textual artefacts*** were written assignments (15% of all artefacts). The proportion of written assignments increased over time and were included by 95% of participants in one or more presentations. Teacher feedback represented 2.9% and exam results 2.0% of all artefacts. Eight % of the presentation entries were reflections unsupported by an artefact. These were categorised as textual evidence.

The most common ***visual artefacts*** were photos or screenshots of the participant alone (selfies) or with peers (group photos), representing 20% of all evidence. The proportion of group photos decreased over time. Group photos were related to classwork (41%), clinical work (33%) and social activities (20%). Problem based Learning (PBL) mechanism diagrams represented 12% of all artefacts and were included by 97% of participants. 71% of PBL mechanism diagrams were created during class. 24% were created at home. PBL mechanism diagrams decreased over time. Selfies represented 8% of evidence and were included by 84% of participants. The frequency of selfies did not change over time. Selfies most often represented clinical work (56%), classwork (19%) and social activities or self-care (17%).

Artefacts representing classwork (non-clinical coursework) decreased over time. 88% of artefacts representing classwork were derived from tutorials or laboratory sessions and 11% were from lectures. The number of artefacts representing clinical activity was higher in second than first year reflecting the increase in clinical teaching time. There were increased home-based activities at Time 2 corresponding to the shift in on-line teaching during the coronavirus pandemic. The proportion of artefacts related to social skills and self-care did not change over time and were included by 73% of participants.

Table 2 provides a summary of the most frequently chosen artefacts and activities at each time point and the proportion of participants who included this type of evidence at any time. See Appendix 2 for further coding details and Appendix 3 for a curriculum timeline.

**Table 2:** Summary of most frequently chosen learning experiences and activities.


ARTEFACT AND ACTIVITY		PROPORTION OF ALL EVIDENCE n (%)*				PARTICIPANTS AT ANY TIME n (%)
		
ARTEFACT	DESCRIPTION	TIME 1 (n = 275)	TIME 2 (n = 296)	TIME 3 (n = 264)	ALL (n = 835)	ALL (n = 37)

Written assignments	Graded written work including essays and reflection tasks	36 (13)	35 (12)	54 (21)	125 (15)	35 (94.6)

PBL mechanism diagram	A flow diagram or concept map relating clinical case to physiological processes prepared before or during PBL tutorials	42 (15)	42 (15)	16 (6)	100 (12)	36 (97.3)

Group photos	Image of a group of students including zoom class screenshots	66 (24)	31 (23)	37 (14)	134 (16)	35 (94.6)

Selfies	Image of the participant alone (photograph or artwork)	23 (8)	16 (5)	31 (12)	70 (8)	31 (83.8)

Activity						

Social activity or self-care (Social)	Related to leisure, sport, social or self-care	29 (11)	25 (8)	19 (7)	73 (9)	27 (73.0)

Classwork	Related to non-clinical campus based or online learning throughout years 1 and 2 including small group tutorials or laboratory sessions and lectures	141 (47)	110 (36)	52 (17)	303 (36)	37 (100)

Homework	Study at home alone or with friends	69 (25)	112 (38)	84 (32)	265 (32)	37 (100)

Clinical activity	Introduction to practical clinical skills training tutorials and visits to General Practices	29 (11)	40 (14)	92 (35)	166 (20)	36 (97.3)


*Only the most common artefacts and activities are included. For each artefact only one activity was assigned. For example, a group photo might be coded as classwork, social or clinical activity.

#### Mixed Model Logistic regression

Mixed model logistic regression assessed the influence of participant (gender, residency, past study, and academic performance at the end of year 2), curriculum (classwork, clinical work, homework) and the timing of the interview on the likelihood of inclusion of the four most frequently chosen artefact types (written assignments, PBL mechanism diagrams, group photos, selfies).

The interview time was a significant contributing factor predicting the choice of written assignments (for Time 3: OR = 1.63, 95% CI [1.02–2.63], p < 0.05), PBL mechanism diagrams (for Time 1: OR = 2.31, 95% CI [1.28–4.16], p < 0.01) and group photos (for Time 1: OR = 2.47, 95% CI [1.44–4.23], p < 0.01). Group photos and PBL mechanisms were more likely to be chosen in first year; written assignments were more likely in second year. Selfies were significantly more likely to represent clinical learning (OR = 4.19, 95% CI [1.02–17.28], p < 0.05), compared with other activities. Group photos more likely represented social and clinical activities and less likely to represent homework (for homework: OR = 0.12, 95% CI [0.27–0.53], p < 0.01).

Participant factors contributed to the models for artefact choice but were not statistically significant. Higher academic performance at the end of second year increased the likelihood for written assignments; lower performance (pass or failing) students were more likely to include of PBL mechanisms than high performance students. Males were more likely to choose written assignments, and females more likely to choose group photos. International students and participants with prior study experience were more likely to include group photos than local students and school leavers respectively.

See Appendix 4 for a summary of the logistic regression models.

### Qualitative analysis of evidence reflections

Four themes were identified in the participants’ reflections across all time points to explain the participants’ artefact choices. These were: Landmarks and Progress, Struggles and Strategies, Connection and Collaboration and Joy for Balance. These themes were represented by different artefacts at each time point. These themes are described below with quotes sampled from different time points across the 2-year period. Quotes are identified according to time of interview: T1 (early first year), T2 (late first year), T3 (middle of second year) and participant identifier (P).

#### Landmarks and progress

Landmarks were first experiences for the participant’s learning journey. These were related to new experiences at any time, reflecting a change in focus or new element of the curriculum. Examples of landmark experiences are the first clinical skills tutorial represented by a group photo at the front door of a clinical school at T1, and ‘first contact’ with cadavers in the anatomy lab recorded by participants at T2. Landmark experiences allowed participants to reflect on their progress over time such as in this reflection associated with a photo of the participant taking blood from a peer: “I remember the first time I cannulated …I still had a bit of an arm tremor which made the task difficult to perform… This is contrasted to me now, with a lot more to learn, but also much more confidence in myself than previously”. P6, T3

#### Struggles and strategies

New experiences were often associated with the struggle to develop new strategies or insights. From the beginning of first year, participants described how they had overcome initial uncertainty about expectations or their competence in the face of novel learning experiences. P11 at T1 describes developing new skills using formative quizzes to meet the challenge of PBL tutorials. The artefact chosen was a screenshot of the quiz results.

“I wasn’t very good, as I had no idea how deep or wide my knowledge of the learning objectives [for PBL] had to be … but through attempting these quizzes … I gradually improved in applying my understanding to answer questions and explain concepts to other people.” P11, T1

Many participants also found early attempts at clinical skills challenging. P31 describes how participation was the secret to clinical skills development in a reflection attached to a selfie in a clinical setting: “…I found that though I sometimes felt nervous, I learnt the most from the experience when I fully participated”. P31, T2

In late year one (T2) and second year (T3), written assignments extended the participants’ research and scholarly writing skills. P16 at T2 explained how one of these assignments had changed his understanding, and confidence in critiquing academic writing: “It imparted me with so much, making me a more competent confident critic and academic writer with a sense of ‘power’, yet humbling me simultaneously in understanding the difficulty it truly is in writing a research paper”.

The theme of struggles and strategies was more prominent in the second year (T3) than the first-year reflections (T1 and T2).

#### Connection and collaboration

Reflections about group learning experiences described how peer interaction calibrated, consolidated, and extended learning. This theme in the reflections was more apparent from the second half of first year (T2) as friendships developed. Working with peers was described as both pleasurable and beneficial in this reflection about a group photo in a social setting: “We get more work done when we study together and it’s nice to have people to bounce ideas off of and asking questions when a concept seems confusing”. P32, T2 P9 at T3, in a reflection without an accompanying artefact, described how working collectively benefitted all: “As different people had different strengths and weaknesses, we were able to elevate the performance of the group as a whole”.

#### Joyful memories and balance

Joy was the predominant theme in evidence related to social and leisure activities. Such activities brought balance to the stresses of study and were included across all time points. “This picture is from last weekend when we all went out for Indian dining… Sometimes when I am bored with just staying in with my laptop, I like to meet my friends and just freshen up by some jokes, going out, playing pool, etc.” P24, T2

Some curricular activities were also included because they were enjoyable, without creating a challenge or changing perspective such as: “During this interactive and fun workshop, … and was an effective way of portraying the effects of disadvantage on not only individuals, but also generations”. P2, T2

This group learning experience was memorable and reinforced what was known.

### Integration of findings

**Figure 1:**
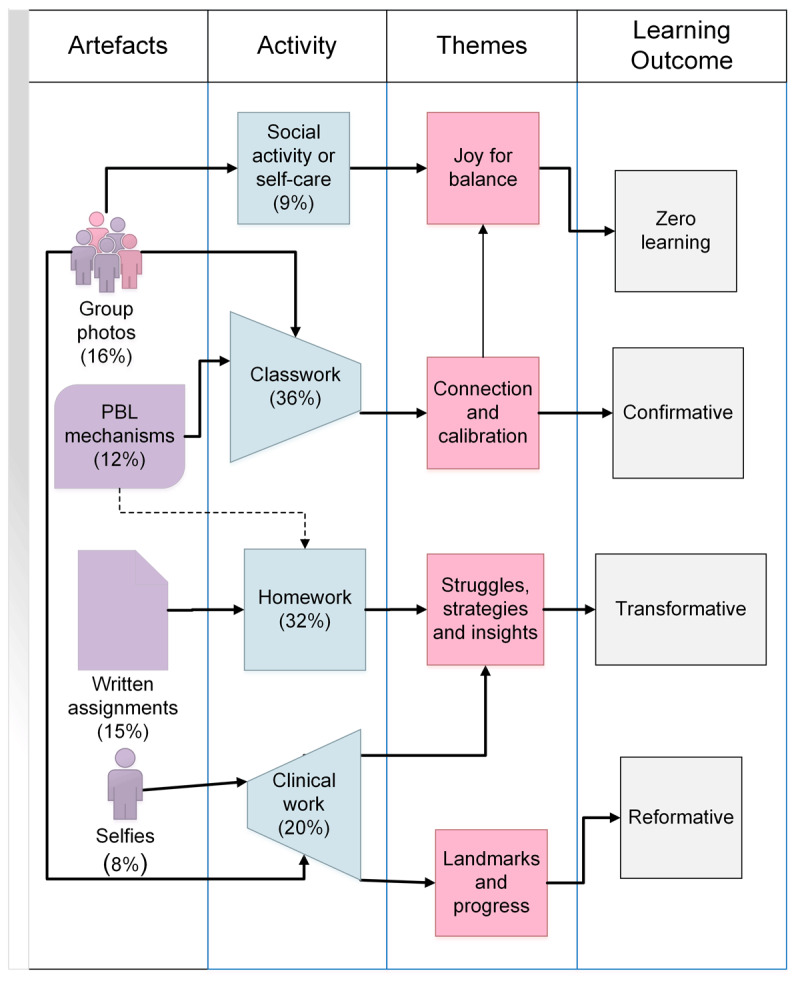
Joint display of integrated results. The first column lists most common types of artefacts and their frequency. These are linked by arrows to the learning activity in the second column and then to the explanatory themes in the third column and the level of TL [21] in the fourth column. The dotted line indicates that a few PBL mechanisms were prepared at home The asymmetric trapeziums in column 2 represent the changing frequency over time. Clinical work increased and classwork decreased.

The most frequent artefact types are mapped to the related learning activities, themes in the reflections and Sterling’s learning levels in Figure 1.

Group photos representing social activities and self-care, were related to reflections encompassing the theme Joyful Memories for Balance. This theme was scattered throughout the presentations over time. International students, female students and students with past study experience were more likely to include group photos, suggesting that belonging was particularly important for these students. Joyful learning experiences were remembered and potentially reinforced prior knowledge.

Group photos and PBL mechanism diagrams were more frequently chosen in Year 1 than Year 2 and were related to reflections about Connection and Collaboration. In these experiences confirmatory learning had taken place in that learning was consolidated through peer interaction. Group learning was also frequently described as enjoyable.

Selfies and group photos documented landmarks and progress: group photos in first year signalled group membership during classwork, and first clinical encounters; selfies in clinical contexts signalled an emerging clinical identity across all times. Identity shifts and group membership are reformative because they change perspective on experience.

Coursework including written assignments and PBL mechanism diagrams during classwork, and artefacts related to clinical work were associated with Struggles and Strategies and descriptions of transformative learning. Increased frequency of these artefacts coincided with the introduction of new curriculum elements creating Landmarks as evidence of progress and explaining the significance of time for artefact choices.

## Discussion

The diverse and changing array of artefacts in the participant’s portfolio presentations reflected the personal nature of response to experience and the myriad opportunities to learn across an integrated curriculum. Artefact types ranged from ‘selfies’ to examples of scholarly writing to represent learning across the curriculum. Artefacts were chosen because they represented landmark experiences, connection with peers, joyful memories or struggles leading to new strategies and insights. Participant factors contributed to artefact choices. This supports and explains the importance of student autonomy in artefact collection [8]. For example, high academic performance and male gender contributed to the likelihood of written assignments, and prior study experience, international student status and female gender contributed to the likelihood of group photos. Time, however, had the most significant impact on the choice of these artefacts. The effect of time was related to changes in the curriculum over the study period.

Novelty explains the significance of time on artefact choice. Over 18 months, the curriculum shifted in focus as new subjects were introduced. Change brings uncertainty, and the need to acquire new skills. As novel challenges were introduced into the curriculum such as PBL, scholarly writing, and clinical skills artefacts were chosen to represent these challenges. For example, the doubling in artefacts representing clinical skills in Year 2 coincided with the introduction of fortnightly simulated clinical procedures training. Over time, participants became more proficient in describing how new academic and clinical experiences exposed knowledge and skill gaps, stimulating the development of new strategies, insights, and goals. New challenges provided the opportunity to recognise and describe TL [17]. This means portfolio tasks should be timed so newly introduced challenges are captured for student reflection and discussion with an advisor.

Not all novel experiences were associated with TL. Novel experiences represented by group photos in first year, and selfies during clinical activities in second year, were indicative of landmarks. The group photos signalled peer connection; the selfies represented an emerging professional identity. New social contexts and identities are reformative according to Sterling by changing perspectives on experience [16]. This provides an opportunity for TL if the new perspective elicits critical reflection. Class photos and the PBL mechanism diagrams also signalled recognition of the importance of group work and relationships for learning [16,19,32], particularly for females, international students, and students with prior study experience. Social interaction, a sense of connection, and dialogue with peers confirms learning by calibrating, challenging and consolidating new ideas and perspectives [16,19].

Students enjoy and value socialising with their peers. Enjoyable experiences also have a place within a medical curriculum as these memorable experiences stimulate curiosity and motivation [33], well-being and contribute to positive self-concept [34]. However, the danger of a focus on affirming and enjoyable experiences, unbalanced by challenge, is an ‘illusion of competence’ without growth [35, p. S51]. Learning experiences, without elements of dissonance, are less likely to stimulate the reflection that allows students to understand the strategies that contributed to the outcome [36]. Without this understanding, performance cannot be replicated or improved, and ‘zero learning’ occurs.

If the purpose of portfolios is to stimulate reflection on learning, to support learning, portfolio design should allow students to represent any experiences that are meaningful, without fear of compromise of progress or loss of face [37]. During student-advisor discussions psychological safety is important because the most valuable lessons are accompanied by disequilibrium and a sense of vulnerability [38]. The advisor’s role is to safely guide critical reflection, drawing on the student’s choice of artefact to recognise new perspectives and goals [36], to deepen the level of learning. The relevance of the portfolio collection to the student will determine the usefulness of the discussion with an advisor. This adds to Driessen’s notion ‘without mentoring, portfolios have no future’ [9, p. 226]. A meaningful collection of artefacts ensures that portfolios, supported by mentors, have a future.

The longitudinal mixed methods design and representative allowed the identification and explanation of patterns in the types of artefacts chosen. The diverse choices in artefacts drawn from different contexts highlights the personal nature of students’ responses to experience and the importance of autonomy in portfolio collections. Photos, written assignments and PBL mechanism diagrams each represented different types of learning and experiences. Our study also shows that timing is a key element of effective portfolio design. Portfolio tasks should be set soon after transitions or the introduction of new challenges to increase the opportunity for reflecting on potentially transformative experiences.

### Limitations

The shift to on-line teaching in response to the coronavirus pandemic coincided with the study period. This is likely to have impacted the range and selection of artefacts. Social relationships were documented with screenshots of peers participating in video conferenced classes and PBL mechanism diagrams were represented by digital whiteboards. The study was from a single institution and portfolio curriculum design limiting the generalisability of our findings. The participants’ artefact choices were constrained by the task requirement and influenced by the intended audience, their advisor.

Further research into the impact of portfolio design, context (pre-clinical, clinical, and post-graduate), mentoring discussions and student perception is needed to further our understanding of the relationship between artefact choices and portfolio-based learning.

## Conclusion

Medical students chose artefacts for their portfolios that represented challenging and/or novel experiences, balanced by experiences that were joyful or fostered peer connection. Challenging, novel and collaborative experiences in any context have the potential to elicit critical reflection on learning and transformation if support is provided. Joyful experiences bring balance and motivation. Time was the strongest predictor of artefact choice. Participant factors contributed to the likelihood of written assignments and group photos. To maximise opportunities for portfolio-based learning, students should be encouraged to draw from the full range of their experiences, to promote supported reflection on learning with an advisor, timed to coincide with the introduction of new challenges and transitions.

## Additional Files

The additional files for this article can be found as follows:

10.5334/pme.1029.s1 Appendix 1. An outline of the Study design including quantitative (QUAN) and qualitative (QUAL) steps.

10.5334/pme.1029.s2 Appendix 2. Summary of coding by artefact type, curriculum subject and learning activity.

10.5334/pme.1029.s3 Appendix 3. The figure below provides a timeline with the most common teaching (above the line) and assessments (below the line) represented by the participants’ artefacts. PPD: Personal and Professional development. Identity map is a PPD assignment. PBL: Problem based learning. ICM: Introduction to Clinical Medicine. O Week: Orientation week.

10.5334/pme.1029.s4 Appendix 4. Mixed Model Logistical Analysis.
