# Supplementary figures and images for: Struggles and Joys: A Mixed Methods Study of the Artefacts and Reflections in Medical Student Portfolios
Source: Perspect Med Educ. 2024 Jan 5;13(1):1–11. doi: 10.5334/pme.1029 (PMC10768569; doi:10.5334/pme.1029)

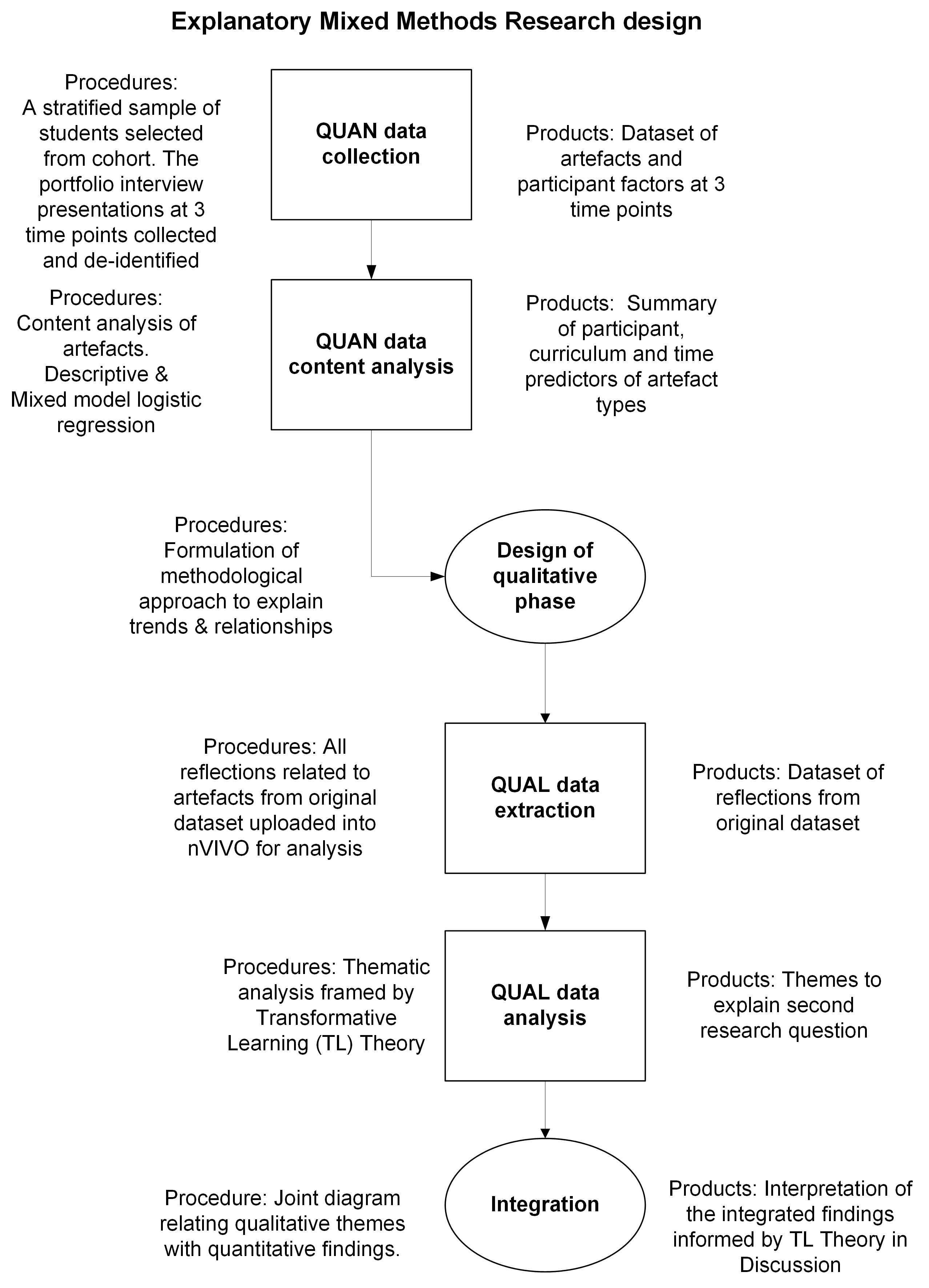

Supplement: Appendix 1. — An outline of the Study design including quantitative (QUAN) and qualitative (QUAL) steps. [file pme-13-1-1029-s1.png]

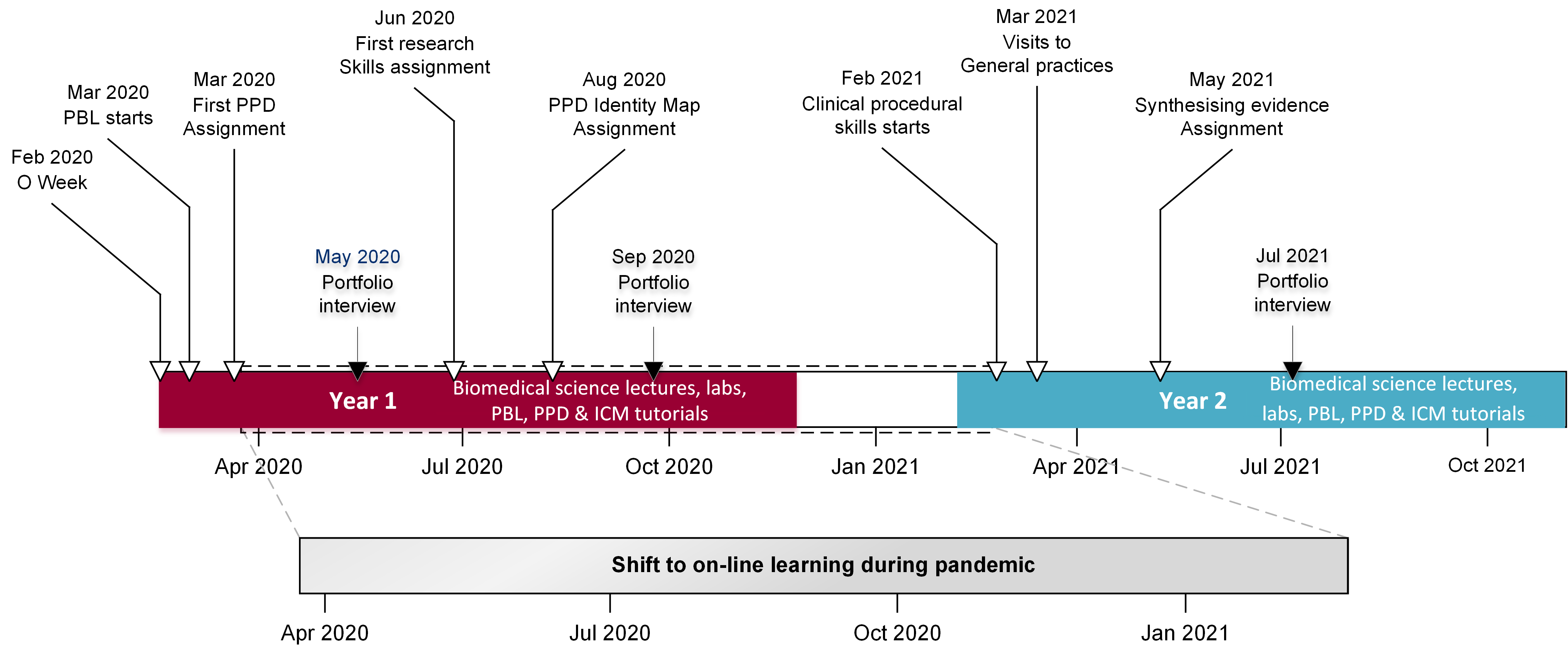

Supplement: Appendix 3. — The figure below provides a timeline with the most common teaching (above the line) and assessments (below the line) represented by the participants’ artefacts. PPD: Personal and Professional development. Identity map is a PPD assignment. PBL: Problem based learning. ICM: Introduction to Clinical Medicine. O Week: Orientation week. [file pme-13-1-1029-s3.png]
