# Supplementary material for: Struggles and Joys: A Mixed Methods Study of the Artefacts and Reflections in Medical Student Portfolios
Source: Perspect Med Educ. 2024 Jan 5;13(1):1–11. doi: 10.5334/pme.1029 (PMC10768569; doi:10.5334/pme.1029)
Supplement: Appendix 2. — Summary of coding by artefact type, curriculum subject and learning activity. [file pme-13-1-1029-s2.pdf]

Appendix 2: Summary of coding by artefact type, curriculum subject and learning activity

		Time 1		Time 2		Time 3		Total	
		n	%	n	%	n	%	n	%
**All evidence**		267	100%	296	100%	261	100%	835	100%
**Format** Textual	Written assignment	36	13%	35	12%	54	21%	125	15%
	Portfolio reflection (without artefact)	9	3%	32	11%	27	10%	68	8%
	Certificate (completed course or on-line module)	4	2%	39	13%	3	1%	46	6%
	Student notes	16	6%	10	3%	18	7%	44	5%
	Other	17	6%	12	4%	12	5%	41	5%
	Lecture slide	5	2%	14	5%	19	7%	38	5%
	Teacher feedback	11	4%	6	2%	7	3%	24	3%
	Quiz or exam results	2	1%	5	2%	10	4%	17	2%
Visual	Peers (group photos)	66	25%	31	10%	37	14%	134	16%
	PBL mechanism diagram	32	12%	30	10%	12	5%	74	9%
	Self	23	9%	16	5%	31	12%	70	8%
	Medical	8	3%	10	3%	16	6%	34	4%
	Physiological process diagram (eg from lecture or text)	10	4%	12	4%	4	1%	26	3%
	Anatomy	9	3%	9	3%	8	3%	26	3%
	Leisure activity	8	3%	10	3%	7	3%	25	3%
	Other	15	6%	10	3%	2	1%	27	3%
	Identity map	0	0%	19	6%	0	0%	19	2%
	Video	8	3%	4	1%	4	1%	16	2%
**Subject**	Personal & professional development (PPD)	82	31%	66	22%	45	17%	193	23%
	Problem based learning (PBL)	76	28%	55	19%	31	12%	162	20%
	Introduction to clinical skills	19	7%	42	14%	88	34%	149	18%
	Extracurricular	22	8%	23	8%	32	12%	77	9%
	Medicine in context	22	8%	15	5%	13	5%	50	6%
	Research Skills	4	1%	24	8%	30	11%	58	7%
	Anatomy	8	3%	31	10%	9	3%	48	6%
	Indigenous health	9	3%	19	6%	7	3%	35	4%
	Basic Science	20	7%	9	3%	3	1%	32	4%
	Other	13	5%	12	4%	6	2%	31	4%
**Activity**	Classwork	141	47%	110	36%	52	17%	303	36%
	Homework	69	25%	112	38%	84	32%	265	32%
	Clinical activity	29	11%	40	14%	92	35%	166	20%
	Social activity or self-care	29	11%	25	8%	19	7%	73	9%
	Group work (in class)	71	27%	46	16%	31	12%	148	18%
	Group work (out of class)	26	10%	8	3%	13	5%	47	6%

	Community visit	3	1%	3	1%	1	0%	7	1%
	Other	5	2%	7	2%	12	5%	24	3%
**Setting**	On-line class	69	26%	125	42%	62	24%	256	31%
	Campus learning	90	34%	13	4%	7	3%	110	13%
	Home	67	25%	89	30%	82	31%	238	29%
	Clinical	16	6%	34	11%	87	33%	137	17%
	On-line other	14	5%	16	5%	13	5%	43	5%
	Social	11	4%	15	5%	10	4%	36	4%
	Community centre	5	2%	4	1%	3	1%	12	1%
	Other	3	1%	0	0%	0	0%	3	0%
