# Supplementary material for: Struggles and Joys: A Mixed Methods Study of the Artefacts and Reflections in Medical Student Portfolios
Source: Perspect Med Educ. 2024 Jan 5;13(1):1–11. doi: 10.5334/pme.1029 (PMC10768569; doi:10.5334/pme.1029)
Supplement: Appendix 4. — Mixed Model Logistical Analysis. [file pme-13-1-1029-s4.pdf]

Appendix 4. Mixed Model Logistical Analysis

Mixed Model Logistical Analysis

Model 1: Written assignments

Variable	β	SE	Sig	OR	95% OR CI Lower	95% OR CI Upper
Intercept	-2.07	1.28	0.11	0.13	0.01	1.57
Time 3	0.49	0.24	0.04	1.63	1.02	2.63
Time 2	-0.11	0.26	0.67	0.90	0.54	1.49
Time 1	0					
Male	0.30	0.21	0.15	1.35	0.90	2.03
Female	0					
High performance	0.25	0.36	0.48	1.29	0.64	2.59
Pass	0.01	0.35	0.97	1.01	0.51	2.03
Fail	0					
Past study	-0.17	0.20	0.40	0.84	0.57	1.26
School leaver	0					

Model 2: PBL Mechanism Diagrams*

Variable	β	SE	Sig	OR	95% OR CI Lower	95% OR CI Upper
Intercept	-2.42	1.17	0.039	0.089	0.009	0.88
Time 1	0.84	0.30	0.006	2.31	1.28	4.16
Time 2	0.76	0.30	0.012	2.13	1.18	3.83
Time 3	0					
Fail	0.31	0.31	0.322	1.36	0.74	2.48
Pass	-0.19	0.24	0.44	0.83	0.52	1.33
High performance	0					
School leaver	-0.19	0.22	0.38	0.83	0.54	1.27
Past study	0					

*Activity not included in model as almost all PBL mechanisms related to classwork

Model 3: Group Photos

Variable	β	SE	Sig	OR	95% OR CI Lower	95% OR CI Upper
Intercept	-2.05	1.39	0.139	0.13	0.008	1.96
Time 1	0.90	0.27	0.001	2.47	1.44	4.23
Time 2	-0.055	0.29	0.85	0.95	0.53	1.68
Time 3	0					
School leaver	-0.26	0.28	0.43	0.80	0.46	1.39
Past study	0					
Domestic	-0.39	0.32	0.23	0.68	0.36	1.27

International	0					
Classwork	0.297	0.58	0.61	1.35	0.43	4.24
Clinical	1.10	0.59	0.06	3.00	0.94	9.54
Homework	-2.12	0.75	0.005	0.12	0.27	0.53
Social	1.34	0.62	0.03	3.81	1.13	12.83
Other	0					
Female	0.25	0.30	0.39	1.29	0.72	2.29
Male	0					
**Model 4: Selfies**						
					95% OR CI	
Variable	β	SE	Sig	OR	Lower	Upper
Intercept	-2.53	1.17	0.030	0.08	0.01	0.79
Time 1	0.131	0.31	0.67	1.14	0.62	2.09
Time 2	-0.24	0.32	0.45	0.79	0.42	1.46
Time 3	0					
Classwork	-0.31	0.74	0.68	0.73	0.17	3.16
Clinical	1.43	0.72	0.048	4.19	1.02	17.28
Homework	-0.67	0.77	0.38	0.51	0.11	2.31
Social	0.93	0.77	0.23	2.52	0.56	11.36
Other	0					
